# Supplementary material for: Pyruvate Dehydrogenase Kinase 1 inhibition mediated oxidative phosphorylation enhancement in cartilage promotes osteoarthritis progression
Source: BMC Musculoskelet Disord. 2023 Jul 20;24:597. doi: 10.1186/s12891-023-06585-6 (PMC10357736; doi:10.1186/s12891-023-06585-6)
Supplement: Supplementary file 1 — Additional file 1. [file 12891_2023_6585_MOESM1_ESM.zip › KEGG.Gsea.1653622432464/index.html]

Index for xtools.gsea.Gsea KEGG.Gsea.1653622432464

### GSEA Report for Dataset 55457

#### Enrichment in phenotype: **OA (10 samples)**

- 73 / 169 gene sets are upregulated in phenotype **OA**- 34 gene sets are significant at FDR < 25%- 19 gene sets are significantly enriched at nominal pvalue < 1%- 29 gene sets are significantly enriched at nominal pvalue < 5%- Snapshot of enrichment results- Detailed enrichment results in html format- Detailed enrichment results in TSV format (tab delimited text)- Guide to interpret results

#### Enrichment in phenotype: **normal (10 samples)**

- 96 / 169 gene sets are upregulated in phenotype **normal**- 23 gene sets are significantly enriched at FDR < 25%- 11 gene sets are significantly enriched at nominal pvalue < 1%- 24 gene sets are significantly enriched at nominal pvalue < 5%- Snapshot of enrichment results- Detailed enrichment results in html format- Detailed enrichment results in TSV format (tab delimited text)- Guide to interpret results

#### Dataset details

- The dataset has 22282 native features- After collapsing features into gene symbols, there are: 12901 genes

#### Gene set details

- Gene set size filters (min=15, max=500) resulted in filtering out 17 / 186 gene sets- The remaining 169 gene sets were used in the analysis- List of gene sets used and their sizes (restricted to features in the specified dataset)

#### Gene markers for the **OA** *versus* **normal** comparison

- The dataset has 12901 features (genes)- # of markers for phenotype **OA**: 5935 (46.0% ) with correlation area 44.8%- # of markers for phenotype **normal**: 6966 (54.0% ) with correlation area 55.2%- Detailed rank ordered gene list for all features in the dataset- Heat map and gene list correlation  profile for all features in the dataset

#### Global statistics and plots

- Plot of p-values *vs.* NES- Global ES histogram

#### Other

- Parameters used for this analysis

#### Comments

- Timestamp used as random seed: 1653622432464

#### Citing GSEA and MSigDB

To cite your use of the GSEA software please reference the following:

- Subramanian, A., Tamayo, P., et al. (2005, PNAS). - Mootha, V. K., Lindgren, C. M., et al. (2003, Nature Genetics).

For use of the Molecular Signatures Database (MSigDB), to cite please reference   
one or more of the following as appropriate, along with the source for the gene set as listed on the gene set page:

- Liberzon A, et al. (Bioinformatics, 2011). - Liberzon A, et al. (Cell Systems 2015).

---

Report: KEGG.Gsea.1653622432464.rpt   by user: ju

xtools.gsea.Gsea [Fri, May 27, '22 11 AM 33]

Website: www.gsea-msigdb.org/gsea
Questions & Suggestions: Contact page
